# Fibrinogen storage disease in a Chinese boy with *de novo* fibrinogen Aguadilla mutation: Incomplete response to carbamazepine and ursodeoxycholic acid

**DOI:** 10.1186/s12876-016-0507-3

**Published:** 2016-08-12

**Authors:** Mei-Hong Zhang, A. S. Knisely, Neng-li Wang, Jing-Yu Gong, Jian-She Wang

**Affiliations:** 1 Department of Pediatrics, Jinshan Hospital, Fudan University, Shanghai, 201508 China; 2 Institute of Liver Studies, King’s College Hospital, Denmark Hill, London, SE5 9RS UK; 3 The Center for Pediatric Liver Diseases, Children’s Hospital of Fudan University, Shanghai, 201102 China; 4 Department of Pediatrics, Shanghai Medical College, Fudan University, Shanghai, 201102 China; 5 Present address: Institute of Pathology, Medical University Graz, Auenbruggerplatz 25, 8036 Graz, Austria

**Keywords:** Fibrinogen storage, Endoplasmic reticulum storage, *FGG*

## Abstract

**Background:**

Fibrinogen storage disease (FSD) is a rare autosomal-dominant disorder caused by mutation in *FGG*, encoding the fibrinogen gamma chain. Here we report the first Han Chinese patient with FSD, caused by *de novo* fibrinogen Aguadilla mutation, and his response to pharmacologic management.

**Case presentation:**

Epistaxis and persistent clinical-biochemistry test-result abnormalities prompted liver biopsy in a boy, with molecular study of *FGG* in him and his parents. He was treated with the autophagy enhancer carbamazepine, reportedly effective in FSD, and with ursodeoxycholic acid thereafter. Inclusion bodies in hepatocellular cytoplasm stained immune-histochemically for fibrinogen. Selective analysis of *FGG* found the heterozygous mutation c.1201C > T (p.Arg401Trp), absent in both parents. Over more than one year’s follow-up, transaminase and gamma-glutamyl transpeptidase activities have lessened but not normalized.

**Conclusion:**

This report expands the epidemiology of FSD and demonstrates idiosyncrasy in response to oral carbamazepine and/or ursodeoxycholic acid in FSD.

**Electronic supplementary material:**

The online version of this article (doi:10.1186/s12876-016-0507-3) contains supplementary material, which is available to authorized users.

## Background

Fibrinogen, synthesized in the liver, is the focal point of the coagulation cascade. The mature circulating molecule is a dimer, with each half composed of 3 polypeptide chains (Aα, Bβ, and γ) encoded by 3 different genes. Defects in these genes can cause hypofibrinogenemia with or without abnormal circulating fibrinogen, resulting in coagulopathy with bleeding and thrombosis as well as in renal-predominant amyloidosis. When mutation in the fibrinogen gamma chain gene (*FGG*) results not only in hypofibrinogenemia but also in accumulation of fibrinogen within endoplasmic reticulum (ER) of hepatocytes and varying degrees of liver disease, fibrinogen storage disease (FSD) can be diagnosed [1].

Since the first report of FSD due to *FGG* mutation [2], FSD has been found in Europe, America, Japan, Saudi Arabia, and Turkey [3–11], but not in China. Data on medical management of FSD are few [10, 12, 13]; carbamazepine (CBZ) and ursodeoxycholic acid (UDCA) may be useful [12, 13]. We report the first Han Chinese patient with FSD confirmed by morphologic studies and molecular analysis and describe his response to CBZ and UDCA administration.

## Case presentatation

A 2-year-old boy was referred for evaluation. He was born to healthy parents at 34 weeks’ gestation (weight 2450 g). At age 8 months, on hospitalization for bronchopneumonia, mild clinical-biochemistry abnormalities suggesting hepatocellular injury (“transaminitis”) were noted; these remained after acute respiratory disease resolved. At age 20 months, on evaluation for epistaxis, mild transaminitis and hypofibrinogenemia were found. Transaminitis and a prolonged coagulation time persisted even after vitamin K1 injection.

The patient weighed 13.0 kg (50–80 percentile); he was 87 cm tall (25–50 percentile). He was not pale. The liver was palpable 3.5 cm below the right costal margin. The spleen was not palpable. Abdominal ultrasonography showed mild hepatomegaly. Complete blood count results were normal. Clinical-biochemistry studies found elevated serum activities of alanine aminotransferase (ALT, 529.6 IU/L; expected < 60), aspartate aminotransferase (AST, 298.2 IU/L; < 60), and γ-glutamyl transpeptidase (GGT, 59 IU/L; < 50). Markers of liver protein synthesis, including total protein (TP, 65 g/L) and albumin (ALB, 49 g/L), were within expected ranges, as were immunoglobulin and ceruloplasmin values. No serologic evidence of active hepatitis A, B, C, or E infection was identified. Profiles of plasma amino acids and urine organic acids were normal. No antinuclear antibodies or antibodies against liver-kidney microsomes (type 1) or smooth muscle could be demonstrated.

Profound hypofibrinogenemia (fibrinogen 0.29 g/L; 2.38–4.98) was found, as were a prolonged prothrombin time (PT, 17.1 s; 9.0–14.5) and activated partial thromboplastin time (APTT, 42.8 s; 25–39). No clinical-biochemistry evidence of hepatobiliary injury or coagulopathy was identified in either parent.

The patient underwent liver core needle biopsy with informed consent of the parents. The biopsy specimen was routinely fixed in phosphate-buffered formalin, processed into paraffin, and sectioned for staining (hematoxylin-eosin [H&E]; periodic acid–Schiff technique with and without diastase predigestion) and immunostaining (antibodies against alpha-1-antitrypsin, alpha-1-antichymotrypsin, and fibrinogen). Fixation for ultrastructural study was not undertaken.

FSD was suspected on clinicopathologic grounds. With the approval of the ethics committee on human research of the Jinshan Hospital of Fudan University and informed consent of the parents, peripheral-blood samples were obtained from the patient and his parents. Genomic DNA was extracted routinely and exons 8 and 9 of *FGG*, which all previous FSD mutations have been located in [14], were amplified by polymerase chain reaction (conditions available on request). Purified PCR products were directly sequenced on an ABI Prism 3730 Genetic Analyzer (Applied Biosystems, Foster City, CA). The oligonucleotide primers used were: Exon 8, forward 5′-AGGGTCAGCATGTATGGTT-3′ and reverse 5′-TCCACTTCCAGTTTCAAAGAA-3′; exon 9, forward 5′-ACTGGCAATGCACTTCGTAA-3′ and reverse 5′-AAAAAGGAAGAAACTTTCAG AGAA-3′. Sequence analysis used BIOEDIT software (North Carolina State University, Raleigh, NC) with NM_000509.4 as reference sequence. Variations were named according to the guidelines of the Human Genome Variation Society. Commercial testing was carried out to exclude the possibility of alternative paternity.

**Fig. 1:**
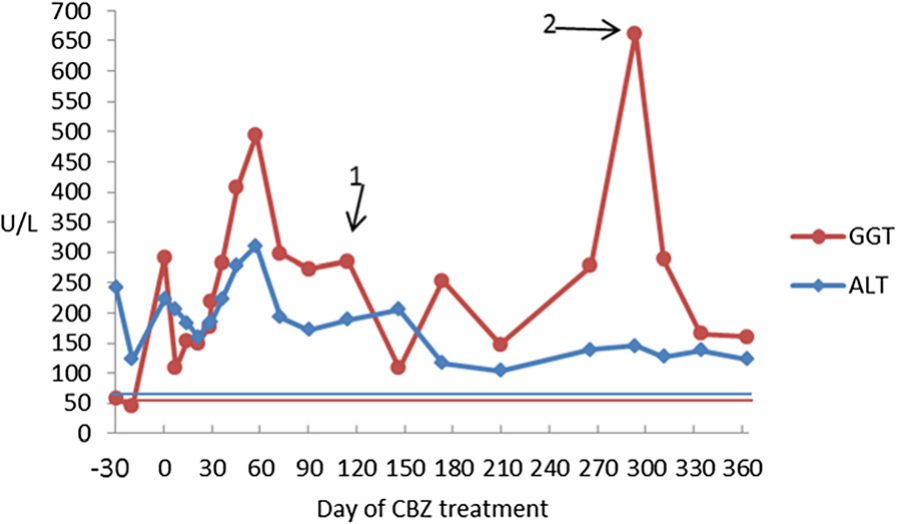
Evolution of ALT and GGT activities after CBZ administration; horizontal lines, upper bounds of expected ALT and GGT values (60 and 50 U/L respectively). 0, X axis: Day immediately before CBZ begun. Arrow 1, serum concentration of CBZ determined (5.59 μg/ml). Arrow 2, ursodeoxycholic acid begun (20 mg kg^−1^ d^−1^)

Guidelines for therapy of FSD are not defined. With the informed consent of the parents, we administered CBZ orally as described [12], with close follow-up. Twice-daily doses began at 25 mg (50 mg/d) and rose gradually to 125 mg (250 mg/d; 19.2 mg kg^−1^d^−1^) in 2 months, with clinical-biochemistry monitoring every 2 to 4 weeks. The dose was maintained for 6 months. Persistently elevated ALT and GGT levels and the worry of CBZ-related toxicity led us to decrease the dose of CBZ gradually from day 240 (Fig. 1). After about 300 days’ administration of CBZ, substantially elevated GGT prompted addition of UDCA (20 mg kg-1 d-1, divided twice), reportedly useful in the management of FSD [13].

**Fig. 2:**
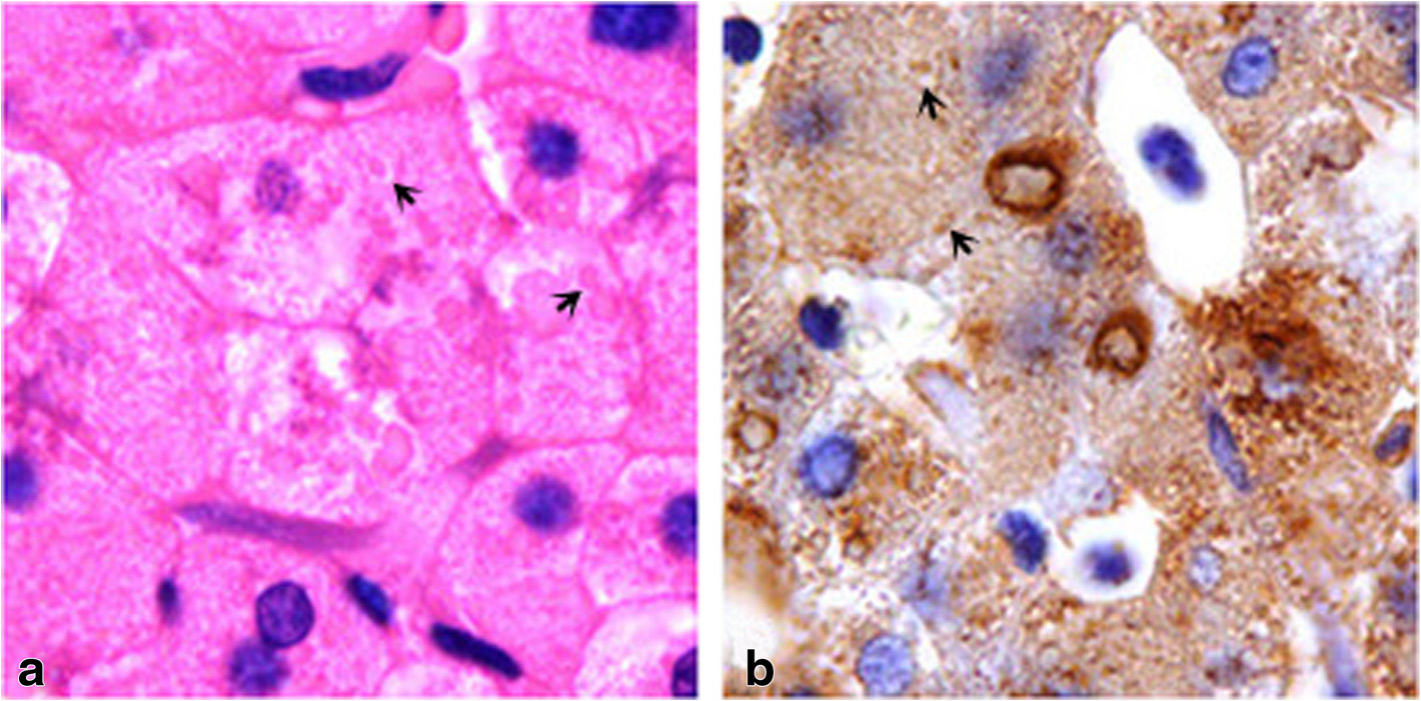
Liver. Palely eosinophilic intracytoplasmic inclusion bodies (**a**, H&E) mark at their margins on immunostaining for fibrinogen (**b**, anti-fibrinogen – diaminobenzidine / hematoxylin). Original magnifications, both images, 1,000×

Mild portal-tract fibrosis and slight chronic inflammation were present, with rare necrotic individual hepatocytes. Hepatocyte cytoplasm was finely granular. Scattered hyaline, eosinophilic inclusion bodies up to 6 μ in diameter were set off by focal cytoplasmic edema (Fig. 2a). These stained only palely on periodic acid–Schiff staining; staining was not altered by diastase pretreatment. On immunostaining, the bodies stained for fibrinogen (Fig. 2b) but not alpha-1-antitrypsin or alpha-1-antichymotrypsin. FSD was diagnosed.

*FGG* sequence analysis in the patient detected heterozygosity for a known disease-causing mutation (c.1201C > T/p.Arg401Trp) (Additional file 1: Supplementary data), predicted to yield fibrinogen Aguadilla [3, 6–8, 10–13]. No mutation was found in either parent. Commercial testing found non-FGG marker inheritance patterns consistent with paternity as declared.

CBZ treatment led initially to a decrease in transaminitis (Fig. 1). A serum concentration of CBZ 4 months after treatment began was 5.59 g/ml. Unfortunately, ALT values had not returned to expected ranges (below upper horizontal line, Fig. 1) on about 300-day follow-up. When serum GGT activity reached 13× upper bounds of expected ranges, ursodeoxycholic acid was added; GGT levels, but not ALT levels, rapidly fell. To date, both ALT and GGT have not returned to ranges expected in health.

During the follow-up period, growth has been normal; side effects of CBZ (e.g., hypoleukocytosis or thrombocytopenia, hematuria) have not been identified on routine whole blood cell count and urinalysis, and neither arrhythmia nor intraocular haemorrhage was revealed by 6-monthly electrocardiography and ophthalmoscopy.

## Conclusion

The rare autosomal-dominant hereditary disorder FSD was first described in German families in 1981 [15]. At this writing, genetically confirmed cases are recorded in 16 families: Information on medical management is scant [10, 12, 13]. Here we report the first histopathologically and genetically documented case of FSD from China, in a Han Chinese boy whose disorder has responded only partially to CBZ and UDCA therapy.

Congenital hypofibrinogenemia can be caused by mutations in *FGA*, *FGB,* or *FGG*. However, only 6 mutations (listed in Table 1, with predicted sequences) in the *FGG* sequence encoding the fibrinogen γ chain residues between 310 and 401 (including signal peptide) are known to cause hypofibrinogenemia and hepatocyte ER storage of abnormal fibrinogen [2–13]. Among them, c.1201C > T (fibrinogen Aguadilla) is repeatedly encountered [3, 6–8, 10–13]. It can be inherited or de novo, that is, not detected in either parent, as our patient, with genetic evidence supporting declared paternity. This site within FGG appears peculiarly susceptible to mutation likely because the site involves a CpG sequence which is subject to hypermutation through methylation and deamidation of C to T [16].

**Table 1:** Reported mutations resulting in fibrinogen storage disease

Name	Nucleotide change	Amino acid change (NM_000509.4)	Amino acid change (originally described, without signal peptide)
Brescia [2]	c.928G > C	p.Gly310Arg	p.Gly284Arg
Aguadilla [3, 6–8, 10–13]	c.1201C > T	p.Arg401Trp	p.Arg375Trp
Anger [4]	c.1115_1129delGAGTTTATTACCAAG	p.G372_Q376del	p.G346_Q350del
AI DuPont [5]	c.1018A > C	p.Thr340pro	p.Thr314pro
Pisa [9]	c.1024G > A	p.Asp342Asn	p.Asp316Asn
Beograd [9]	c.1174G > A	p.Gly392Ser	p.Gly366Ser

Most FSD patients have been asymptomatic, with incidental diagnosis during routine laboratory examination. Aside from epistaxis, our patient had no obvious symptoms; his disorder was suspected only when clinical-biochemistry abnormalities were noted and coagulation parameters were assessed. Histological examination of a liver-biopsy specimen found in hepatocellular cytoplasm inclusion bodies that marked immunohistochemically for fibrinogen.

However, some individuals who exhibit only hypofibrinogenemia harbor mutations that in family members are associated with FSD [2, 8–11]. Retention of abnormal proteins within ER may, but need not, lead to liver disease; this has been attributed to variation in ER degradation [17]. Background differences may influence response to treatment. Two patients with FSD (fibrinogen Aguadilla) who were given the autophagy-enhancing agent CBZ responded well, with rapid normalization of serum ALT activity (35 and 26 days) [13]. However, in our patient gradually increased doses of CBZ, as described, reduced transaminitis without effecting normalization. The transient elevation of GGT activity during CBZ administration may be related to toxicity of CBZ [18], although this has not been seen in FSD patients before. Given the absence of other liver disease, we ascribe incomplete response in our patient to idiosyncrasy.

Treatment for 36 months with UDCA and vitamin E also has been reported to normalize patient transaminase-activity values [13]. We gave UDCA in response to the GGT activity spike and to persistently abnormal ALT activity after approximately 290 days of follow-up. GGT activity decreased sharply after UDCA was begun, but to date (about 70 days of follow-up at this writing after UDCA begun) ALT activity is essentially unchanged. The results may be associated with the short treated days and need further observation.

We present our experience with FSD and its treatment to advise colleagues that Han Chinese patients may develop FSD; our patient is the first described from this ethnic group, but others will likely be seen. We also wish to temper possible enthusiasm for CBZ and UDCA treatment of FSD by reporting that in our hands its success has been, to date, incomplete.

## Abbreviations

ALT, alanine aminotransferase; CBZ, carbamazepine; ER, endoplasmic reticulum; FGG, fibrinogen gamma chain gene; GGT, γ-glutamyl transpeptidase; UDCA, ursodeoxycholic acid.

## Additional file

Additional file 1: Supplementary material: Figure. The information of the mutation in the fibrinogen gamma chain gene (*FGG*) of the patient. (BMP 685 kb)
