# Supplementary figures and images for: Fibrinogen storage disease in a Chinese boy with de novo fibrinogen Aguadilla mutation: Incomplete response to carbamazepine and ursodeoxycholic acid
Source: BMC Gastroenterol. 2016 Aug 12;16:92. doi: 10.1186/s12876-016-0507-3 (PMC4981954; doi:10.1186/s12876-016-0507-3)

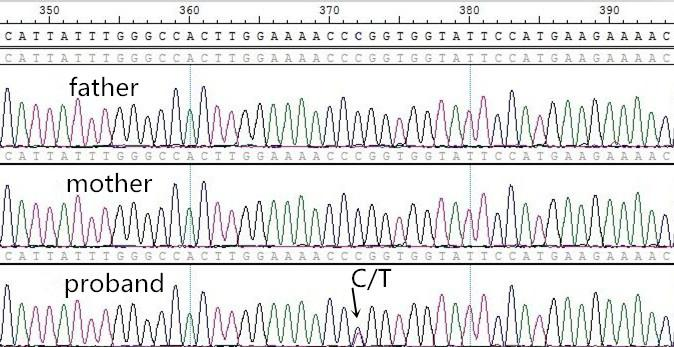

Supplement: Supplementary file 1 — Supplementary material: Figure. The information of the mutation in the fibrinogen gamma chain gene (FGG) of the patient. (BMP 685 kb) [file 12876_2016_507_MOESM1_ESM.bmp]
